# Fabrication of Chitosan Nanofibers Containing Some Steroidal Compounds as a Drug Delivery System

**DOI:** 10.3390/polym14102094

**Published:** 2022-05-20

**Authors:** Mohamed Gouda, Mai M. Khalaf, Saad Shaaban, Hany M. Abd El-Lateef

**Affiliations:** 1 Al Bilad Bank Scholarly for Food Security in Saudi Arabia, The Deanship of Scientific Research, The Vice Presidency for Graduate Students and Scientific Research, King Faisal University, Al-Ahsa 31982, Saudi Arabia; mmkali@kfu.edu.sa (M.M.K.); sibrahim@kfu.edu.sa (S.S.); 2 Department of Chemistry, College of Science, King Faisal University, Al-Ahsa 31982, Saudi Arabia; 3 Department of Chemistry, Faculty of Science, Sohag University, Sohag 82524, Egypt; 4 Department of Chemistry, Faculty of Science, Mansoura University, Mansoura 35516, Egypt

**Keywords:** chitosan nanofibers, electrospinning technique, drug delivery, steroidal compounds

## Abstract

A novel drug delivery system based on chitosan nanofibers containing some steroidal derivatives was developed using an electrospinning process. Oxazolines and aziridines from the cholestane series of steroidal epoxides were successfully synthesized and characterized by elemental analysis, Fourier transforms infrared spectroscopy (FTIR), proton nuclear magnetic resonance (^1^HNMR), and mass spectroscopy (MS). Steroidal-compound-loaded chitosan (ST-CH) nanofibers were fabricated using the electrospinning technique in the presence of polyvinylpyrrolidone (CH/PVP). The electrospun nanofibers were characterized by scanning electron microscopy (SEM). The swelling degree of the electrospun nanofibers and their steroidal compound release performance were studied as well. Furthermore, their antibacterial activity against gram-positive (*Staphylococcus aurous*) and gram-negative bacteria (*Escherichia coli*) was evaluated. The experimental data revealed that identical and bead-free nanofiber mats loaded with 10 Wt. % of synthesized steroidal derivatives had been obtained. The FTIR spectrum proved that no change occurred in the chitosan structure during the electrospinning process. The synthesized nanofiber mats showed a high swelling degree and a burst release of steroidal compounds after 2 h doping in phosphate buffer saline. In addition, the electrospun nanofibers containing 3β-chloro-N-amido-5α-cholestano-aziridine and those containing 3β-acetoxy-N-amido-5α-cholestano-aziridine were the most active, with activity indices of 91 and 104% in the case of S. aureus and 52% and 61% in the case of *E. coli*, respectively. The release mechanism by CH/PVP of the drug samples was studied based on the charge density and diffusion controlled factors. The oxazoline derivatives release mechanism from CH/PVP was evaluated by applying the suppositions of the Ritger-Peppas kinetic model and by estimating the transport exponent; the latter revealed the involvement of the solvent diffusion and chain relaxation processes. Tailored steroidal loaded-chitosan (ST-CH) nanofibers are expected to be feasible and efficient drug delivery systems.

## 1. Introduction

Chitosan is a naturally occurring polymer of deacetylated chitin, which is the second most plentiful polysaccharide, occurring in crab, shrimp shells, fungal mycelia, and some mushrooms (zygote fungi). Furthermore, chitosan is the only pseudo-natural cationic with plenty of primary amines. It exhibits remarkable biocompatibility, biodegradability, adsorption activity, and antimicrobial ability [1]. Chitosan is a linear polysaccharide which consists of indiscriminately scattered β-(1-4)-linked D-glucosamine (deacetylated unit) and N-acetyl-D-glucosamine (acetylated unit). Furthermore, chitosan has one amino group and two hydroxyl groups in the repeating unhydroglucose unit [1,2,3,4,5,6,7,8].

Chitosan is a semi-crystalline polymer which can exist in an amorphous state, depending on its degree of acetylation, spreading of the acetyl groups along the carbohydrate chain, and its preparation route [9]. In addition, chitosan is insoluble in water but soluble in acidic aqueous media due to the protonation that occurs in the –NH_2_ group which exists at the C-2 position of the unhydroglucose unit. Through this mechanism, the polysaccharide is transformed into a polyelectrolyte. The solubility of chitosan in acetic acid can provide an ionically conductive solution due to the presence of protons from the acid [10].

Due to the biocompatibility of chitosan, it has been used as a hemostatic agent from which blood anticoagulants and antithrombogenics have been formed and processed into different forms such as gels, membranes, beads, microparticles, nanofibers, scaffolds, and sponges [11,12,13,14,15].

Further benefits of chitosan are its ease of use, low cost, high biocompatibility, biodegradability, antimicrobial qualities, ease of chemical modification, and excellent film-forming capability [16].

Furthermore, owing to its weak acidic pKa, chitosan can simply be bound with a negative charge surface containing a bio-membrane. This bio-bonding agent can be applied to improve the efficacy of the desired drug delivery system. The adhesion characteristics of chitosan improve the bonding with drug compounds, extend the time of its discharge, and increase the bio application of the drug [17]. Chitosan has been synthesized on a nanoscale, i.e., nanoparticles, for drug delivery [13]. In medical applications, nanofibers are used as tissue engineering scaffolds and for drug delivery and wound dressing [14]. A wound dressing material must be biocompatible, biodegradable, accelerate the healing process, and have high porosity for respiration [15,18].

Nanofibers were developed using template synthesis, drawing, phase separation, electrospinning, self-assembly, and so forth [19]. Among these methods, electrospinning is particularly versatile for the fabrications of micro and nanofibers [14,20]. Electrospun nanofibers have a high surface area to volume ratio, oxygen permeable porosity, and different pore sizes; as a wound dressing material, they initiate cell adhesion, migration, and fibroblast proliferation [21].

Recently, the fabrication of a chitosan nanofiber composite was reported from pure or blended materials. In the latter case, synthetic polymers were used, such as polyvinyl alcohol, polyethylene terephthalate, polyethylene oxide, polycaprolactone, polylactic acid, nylon-6, and others, to enhance the nanofibers in terms of their mechanical, biocompatible, and antibacterial properties [22].

Steroids are novel bioactive phytochemicals with therapeutic potential and no or significantly reduced side effects. They are a special type of chemical molecule that may be found in animals and plants. Steroidal drugs are used to treat inflammatory conditions; however, they have serious negative effects when taken long-term [23].

**Scheme 1:**
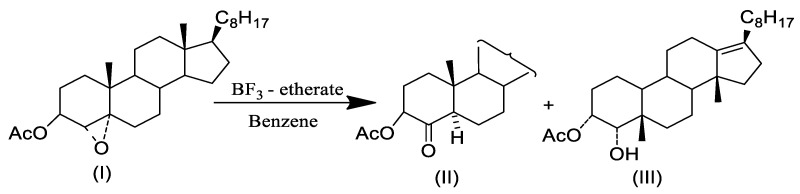
Regio- and stereoselective cleavage of 3α-acetoxy-4α,5-epoxy-cholestane, catalyzed by BF_3_. Et_2_O, steroids.

In this study, the method described in [24] for carrying out ring-opening and fluoridation reactions of steroidal epoxide were used. The literature describes the reaction of 3α-acetoxy-4α,5-epoxy-cholestane (**I**) with BF_3_-etherate in benzene, yielding 3α-acetoxy-5a-cholestan-4-one (**II**), along with a rearranged product of 3α acetoxy-4 α-hydroxyl–compound (**III**) Scheme 1 [25].

The present research was undertaken with a view to developing a novel drug delivery system. This involved fabricating chitosan nanofiber mats loaded with synthesized steroidal compound drugs. The electrospinning technique was applied for this synthesis. The impact of electrospinning on the bioactivity of the drug and the sustainability of the release behavior from the electrospun nanofiber mats were studied. In addition, the antibacterial activity of the drug-loaded chitosan nanofiber mat vis-a-vis neat chitosan nanofiber mats is described.

## 2. Experimental Methods

### 2.1. Materials

Cholesterol was obtained from BDH biochemical. Cholesteryl chloride 97%, benzamide 99%, semicarbazide hydrochloride 99%, ethanol absolute 99.8%, glacial acetic acid 99.5%, acetic anhydride 99%, pyridine 99.5%, diethyl ether 99.8%, chloroform 99.0–99.4%, N, N-dimethylformamide 99.8% and petroleum ether were supplied by Sigma Aldrich, USA. Acetamide 99%, acetone, aluminum chloride anhydrous 98%, benzoyl peroxide 98%, sodium hydroxide 98.0–100.5%, and sodium bicarbonate 99.3% were purchased from Fluke company, Germany. Other chemicals were used as laboratory reagents. Chitosan (85% deacetylated), with an average molecular wt. of 100,000 g/mol, was obtained from Sigma Aldrich. The intrinsic viscosity [η] was measured in a mixture of 0.1 M acetic acid and 0.2 M sodium chloride at 25 °C. Polyvinyl pyrrolidone (PVP) (Mw 900,000 g/mol) was supplied by Sigma Aldrich. All samples were used without further purification.

### 2.2. Synthesis of 3β-Methoxy-5α-cholestano-2′-methyl-2-oxazoline (**V**), 3β-Chloro-5α-cholestano-2′-methyl-2-oxazoline (**VI**), 3β-Methoxy-N-amido-5α-cholestano-aziridine (**VII**), and 3β-Chloro-N-amido-5α-cholestano-aziridine (**VIII**)

**Scheme 2:**
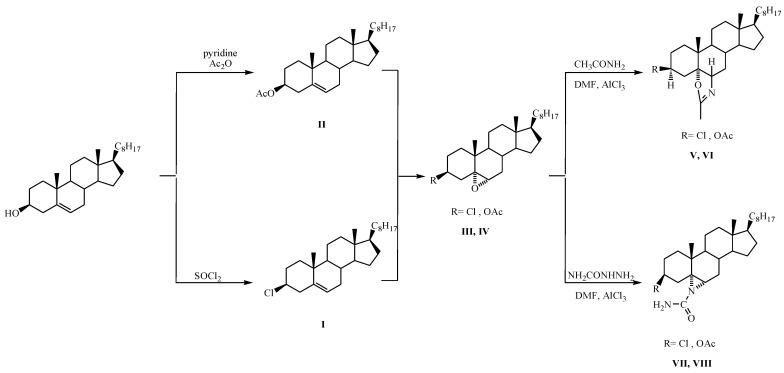
Synthesis of steroidal oxazoline [(**V**),(**VI**)] and aziridine derivatives [(**VII**),(**VIII**)].

The synthesis processes are presented in the following schematic diagram (Scheme 2).

#### 2.2.1. Synthesis of 3β-Chlorocholest-5-ene (**I**)

At room temperature, 75 mL of freshly purified SOCl₂ (thionyl chloride) was added slowly to 100 g of cholesterol. An energetic reaction ensured the creation of gaseous products. The mixture was heated at 323–333 K in a water bath for 60 min and then decanted by stirring onto crushed ice. The obtained compound was filtered under vacuum, and then washed many times with iced water and air-dried. Recrystallizations of the raw product from acetone yielded 5.50 g of the desired compound (m.p. 95–96 °C).

#### 2.2.2. Synthesis of 3β-Acetoxycholest-5-ene (**II**)

A mixture of pyridine (75.0 mL, freshly distilled over potassium hydroxide), 50.0 g of cholesterol, and 50 mL of acetic anhydride were heated in a water bath for 120 min. The resulting brown solution was stirred into a crushed ice–water mixture. A light brown solid was produced, which was then air-dried. The crude product recrystallized from acetone, yielding 45.0 g of the compound (m.p. 113–115 °C; reported m.p. 116 °C).

#### 2.2.3. Synthesis of 3β-Acetoxy 5,6α-epoxy-5α-cholestane (**III**)

First, 10 g of 3β-Acetoxycholest-5ene (**II**) in 100 mL chloroform was pickled with per benzoic acid solution (1.1-mole equivalent) and left for 20 h at a temperature of −8 °C. Subsequently, the blend was washed consecutively with an ice-cold 5% solution of NaHCO_3_, 5% solution of Na_2_S_2_O_3_·5H_2_O, and finally, with water. Solvent evaporation provided an oil that was chromate-graphed over a silica gel column. The substance was then rinsed with petroleum ether-ether (10:1) to produce a solid product that was recrystallized from acetone as needles, yielding give 8.0 g epoxide (**III**) (m.p. 96–97 °C; reported m.p. 97 °C).

#### 2.2.4. Synthesis of 3β-Chloro-5,6α-epoxy-5α-cholestane (**IV**)

First, 11.0 g of 3β-Chloro cholest-5ene (**I**) in 100 mL chloroform was dried with a per benzoic acid solution (1.1 mol equivalent) and left for 20 h at a temperature of −8.0 °C. Subsequently, the mixture was washed sequentially with an ice-cold 5% solution of NaHCO_3_, 5% solution of Na_2_S_2_O_3_·5H_2_O, and finally, with water. Solvent evaporation yielded compound (**IV**) as an oil that was crystallized from acetone as 8.10 g of needles (m.p. 88.0–89.0 °C; reported m.p. 90.0–91.0 °C).

#### 2.2.5. Synthesis of 3β-Chloro-5α-cholestano-2′-methyl-2-oxazoline (**V**)

First, 2.0 g of compound (**IV**) (3β-Chloro-5,6α epoxy-5α-cholestane) dissolved in 50 mL DMF, was treated with 1.0 g of acetamide in the presence of AlCl_3_ anhydrous as a catalyst. Subsequently, the mixture was analyzed by thin-layer chromatography (TLC); the solvent and the residue (1.62) were column chromatographed over 35.0 g of silica gel.

#### 2.2.6. Synthesis of 3β-Acetoxy-5α-cholestano-2′-methyl-2-oxazoline (**VI**)

First, 2.0 g of compound (**II**) (3β-Acetoxy-5,6α epoxy-5α-cholestane) and 1.0 g of acetamide were dissolved in 50 mL of DMF. The mixture was heated under reflux in the presence of anhydrous AlCl_3_ as a catalyst. TLC was used to monitor the progress of the reaction. Upon completion of the reaction, the solvent was evaporated under condensed pressure. The produced compound was removed with ether. The ethereal layer was washed many times with water and dried over anhydrous Na_2_SO_4_. Removal of the solvent yielded oil (1.65 g) (**VI**), which was chromatographed over silica gel (40.0 g).

#### 2.2.7. Synthesis of 3β-Chloro-N-amido-5α-cholestano-aziridine (**VII**)

First, 3β-chloro-5,6α-epoxy-5α-cholestane was dissolved in DMF and treated with semi-carbazide in the presence of anhydrous AlCl_3_ as a catalyst under reflux for 8 h. The reaction mixture, after column chromatography over silica gel, yielded the desired compound (m.p. 132 °C).

#### 2.2.8. Preparation of 3β-Acetoxy–N-amido-5α-cholestano-aziridine (**VIII**)

A mix of 1.5 g semi-carbazide and 1.5 g 3β-acetoxy-5,6α-epoxy-5α-cholestane were dissolved in 25 mL DMF in the presence of AlCl_3_ anhydrous as a catalyst and refluxed for 8 h. The reaction progress was followed with TLC. Upon completion, the reaction blend was dried over anhydrous Na_2_SO_4_. Following the evaporation of the solvent, the remaining compound (1.30 g) was column chromatographed over 30.0 g of silica gel.

### 2.3. Fabrication of Steroidal Compounds Loaded-Chitosan/Polyvinyl Pyrrolidone (ST-CH/PVP) Nanofibers

Chitosan (CH) solution was prepared by dissolving (5wt %) of chitosan in 90% glacial acetic acid. Solutions with constant mass ratios (1:3) gm% of CH and PVP, respectively, were prepared by dissolving 5% chitosan (*w*/*v*) and 15% PVP (*w*/*v*) in glacial acetic acid 90% (*v*/*v*). The prepared solutions of CH/PVP were then mixed with 10wt % in petroleum ether separately; 3β-Chloro-5α-Cholestano-2′-methyl-2-oxazoline (**V**), 3β-acetoxy-5α-Cholestano-2′-methyl-2-oxazoline (**VI**), 3β-chloro-N-amido-5α-cholestano-aziridine (**VII**) and 3β-acetoxy–N-amido-5α-cholestano aziridine (**VIII**) were named as samples A, B, C, and D, respectively. All prepared solutions were stirred with a homogenizing ultrasonic stirrer for 30 min. Electrospinning of the prepared solutions was performed according to the method reported in [26] using a Nanofiber Electrospinning (NEU-010) Unit, Kes Kato Tech Co., Ltd., Kyoto, Japan. In the electrospinning procedure, a high-electric potential was applied to a droplet of every prepared solution at the syringe needle tip (with an internal diameter of 0.15 mm). High-electrical energy (30 kV) was applied to the accumulating target drum using a high-voltage control source. The prepared solution was pumped by syringe pump with a polymer feeding rate of 1.0 mL/h. The electrospun nanofibers were formed on a target drum at a distance from the syringe tip of 15.0 cm. The rotating speed of the rotational target drum was kept at 40.0 ± 5 r/min. The electrospinning method was completed under laboratory conditions (i.e., relative humidity = 71 ± 3%; temperature = 25 ± 1 °C).

### 2.4. Characterization

#### 2.4.1. Characterization of the Synthesized Steroidal Oxazoline and Aziridine

The melting points of the aforementioned compounds were determined using a melting point device. FTIR spectra were attained with a Cary 830 FTIR (Agilent Technologies, Santa Clara, CA, USA) spectrophotometer; FTIR values are reported in cm^−1^. The ^1^HNMR spectra were recorded using a CDCl_3_ NMR Bruker (400 MHz) spectrometer (Bruker Biospin AG, Dresden, Germany) with Tetramethylsilane as the interior standard; the results are reported in ppm (o). Mass spectra were measured using a GC–MS (Shimadzu, Kyoto, Japan) with an AOC 20i auto-injector. The TLC plate was coated with silica gel and sprayed with 20% perchloric acid solution. Next, 20.0 g of silica gel was utilized for 1.0 g of the material in column-chromatography. Petroleum ether was used (b.p. 40–60 °C). Finally, anhydrous Na_2_SO_4_ was utilized as the dehydrating agent.

#### 2.4.2. Characterization of Electrospun Nanofibers

The electrospun nanofibers were characterized using scanning electron microscopy (field emission scanning electron microscope Joel, SM7600F-USA, Pleasanton, CA, Canada).

### 2.5. Degree of Swelling

The swelling degree of the electrospun mats was determined by a gravimetric method. The electrospun mats were impregnated with phosphate-buffered saline (pH = 7.4) at 25 °C for 24 h. The immersed nanofiber samples were taken out, the extra surface water on the samples was removed with filter paper, and the swollen nanofibers were weighed. The ratio of swelling was calculated using the following Equation (1):

(1) $$\mathrm{Swelling}\;\mathrm{ratio}/\%=\left[\frac{W_{s}-W_{0}}{W_{0}}\right]\times 100$$

where *W_s_* indicates the weight of the swelled mat and *W*_0_ indicates the weight of the mat in dry form after 24 h impregnation in phosphate-buffered saline.

### 2.6. Release Study

UV-Visible (UV–Vis-NIR UV 3600-spectrometer Shimadzu Company, Kyoto, Japan) absorbance was considered as an indicator of the release of steroidal oxazoline and aziridine derivatives from electrospun mats. The dehydrated, electrospun, extract-loaded-nanofibrous mats were first divided into 12 × 12 cm^2^ squares and the extracted compound was estimated as a function of the weight mat. The drug loading into CH-PVP nanofibres was 12 mmole/100 gm CH-PVP from each sample, i.e., A, B, C, and D. The specimens were placed in individual vessels containing 50 mL buffer saline phosphate (pH = 7.40), and the vessels were incubated at 310 K. Release experiments were performed by dispersing 200 mg of the ST-CH nanofibers in 50 mL of the buffer solution. After a certain time, 5 mL of the buffer was replaced with the same volume of fresh buffer to maintain a constant volume. A mixture of 3 mL methanol and 2 mL distilled water was prepared and added to each buffer to determine the absorbance by UV-Visible spectroscopy at 300 nm. A standard calibration curve was applied to measure the concentration of the free steroidal oxazoline and aziridine derivatives. The percentage of the released steroidal oxazoline and aziridine derivatives was then calculated based on the initial weight of these compounds incorporated in the electrospun nanofiber mats. The results were plotted over time, up to 60 h.

### 2.7. Antibacterial Activity

The antibacterial activity of the electrospun CH/PVP nanofibers with and without steroidal oxazoline and aziridine derivatives was evaluated against *Staphylococcus aureus* gram-positive and *Escherichia coli* gram-negative bacteria using an agar well diffusion assay, as described in [27,28]. Briefly, the antibacterial strains were seeded in Petri dishes containing 20.0 g of dextrose agar nutrient, 5 g peptone, and 3 g of the beef extract. Next, 1 mM of the electrospun CH/PVP nanofibers was prepared in DMSO. Standard paper discs (5.0 cm) were autoclaved and sterilized. Subsequently, 20.0 µL of the nanofibers were added to the paper discs and placed inside the Petri dishes. The antibacterial drug ampicillin was used as a standard. The dishes were incubated at 30 °C for one day. The clear zones (in mm) were measured and the activity index percentage (A%) was calculated according to Equation 2. Images of the Petri dishes are presented in the support materials.

(2) $$\mathrm{Activity}\;\mathrm{Index}\;/\;\%=\frac{\mathrm{The}\;\mathrm{diametre}\;\mathrm{of}\;\mathrm{the}\;\mathrm{nanofibers}\;\mathrm{inhibition}\;\mathrm{zone}}{\mathrm{The}\;\mathrm{diametre}\;\mathrm{of}\;\mathrm{ampicillin}\;\mathrm{inhibition}\;\mathrm{zone}}\times 100$$

## 3. Results and Discussion

### 3.1. Characterization of Synthesized Steroidal Oxazoline and Aziridine Derivatives

#### 3.1.1. Characterization of 3β-Chloro-5α-cholestano-2′-methyl-2-oxazoline (**V**)

**Scheme 3:**
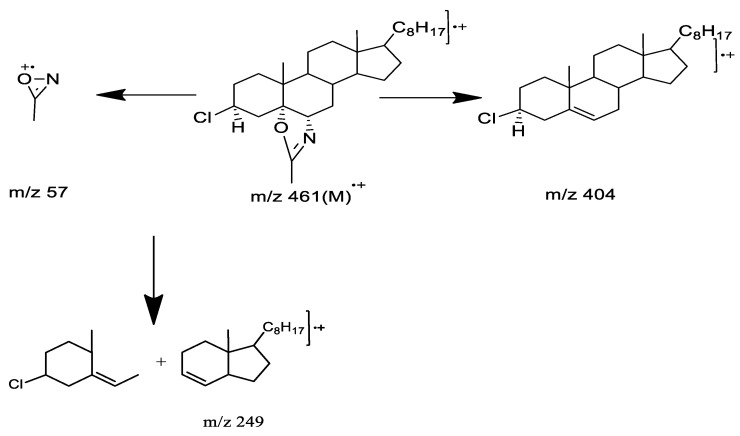
Fragments of mass spectra.

The desired m.p. of 110 °C was confirmed for C_29_H_48_NOCl. As shown in Table 1 and Figure S1, the mass spectrum of the compound presented molecular ion peaks *m*/*z* at 461 (M+), followed by important fragmentations at *m*/*z* 404 and *m*/*z* 57, as shown in Scheme 3.

#### 3.1.2. Characterization of 3 β-Acetoxy-5α-cholestano-2′-methyl-2-oxazoline (**VI**) 

**Scheme 4:**
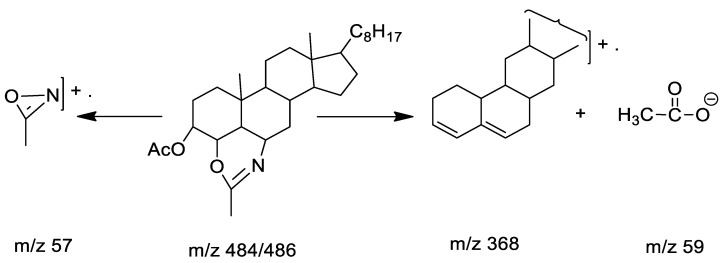
Mass spectra fragmentations.

Table 2 and Figure S2 (Supplementary Materials) characterize 3β-Acetoxy-5α-cholestano-2′-methyl-2-oxazoline (**VI**). The compound was obtained as a semi-solid and analyzed as C_31_H_51_NO_3_. The mass spectrum of the compound revealed a molecular ion peak at *m*/*z* 484/485 (M+), as well as important fragmentations at *m*/*z* 368, *m*/*z* 57, *m*/*z* 59. Fragmentations of lower masses as shown in Scheme 4.

#### 3.1.3. Characterization of 3β-Chloro-N-amido-5α-cholestano-aziridine (**VII**)

**Scheme 5:**
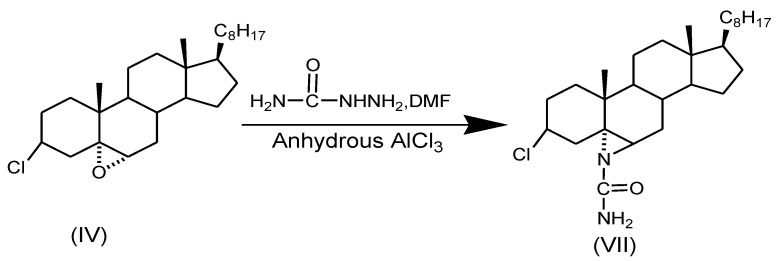
Synthesis of 3β-chloro-N-amido-5α-cholestano-aziridine.

Table 3 and Figure S3 (Supplementary Materials) characterize this compound (m.p. 132 °C) as 3β-chloro-N-amido-5α-cholestano aziridine (**VII**), i.e., C_28_H_47_N_2_OCl. The synthesis process is described in Scheme 5.

#### 3.1.4. Characterization of 3β-Acetoxy-N-amido-5α-cholestano aziridine (**VIII**)

**Scheme 6:**
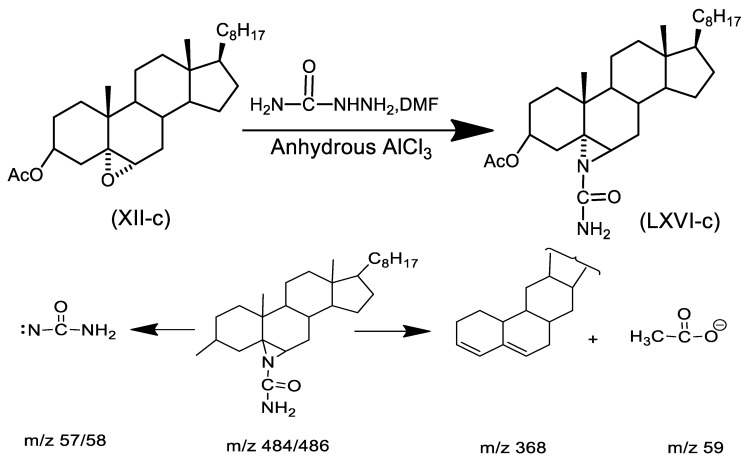
Fragments of mass spectra.

Table 4 and Figure S4 (Supplementary Materials) characterize this compound (m.p. 177–178 °C). The mass spectrum of the compound revealed molecular ions at *m*/*z* 484/486 (M+), followed by some significant fragment peaks at *m*/*z* 368/369, *m*/*z* 57 (NH_2_CO–N), *m*/*z* 59 (M+-CH_3_COO). Lower fragmentation mass peaks are shown in Scheme 6.

### 3.2. SEM Characterization of Electrospun Nanofibers

**Figure 1:**
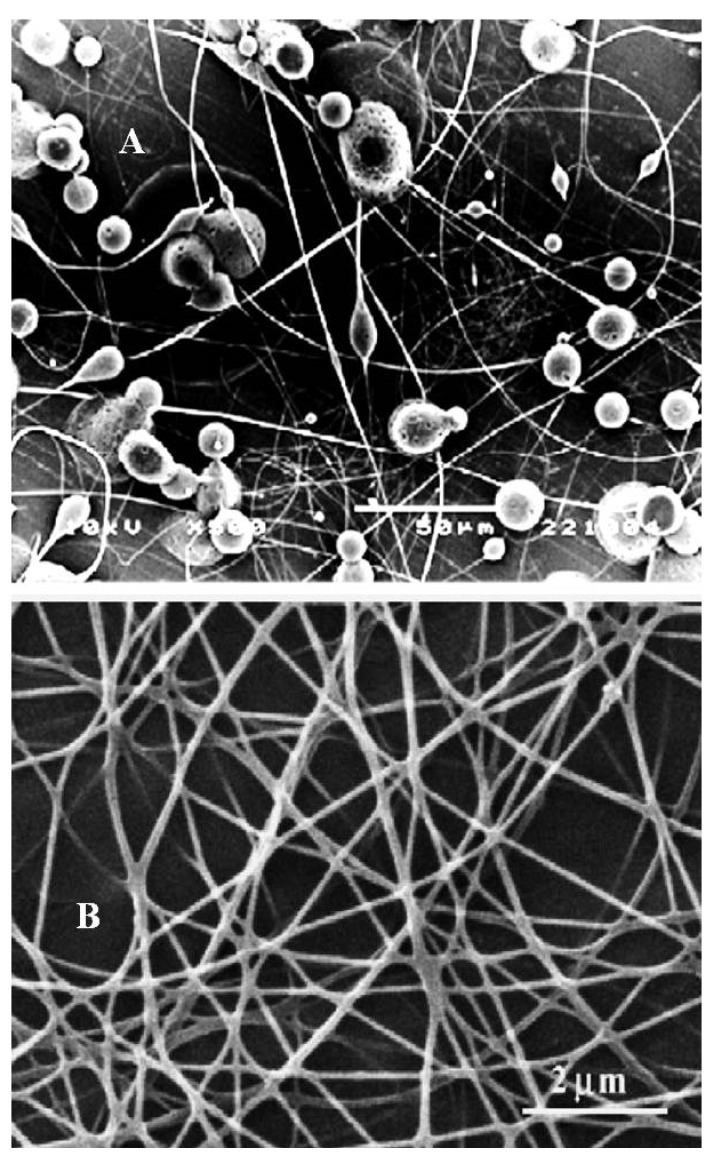
SEM image of electrospun pure chitosan 5% in 90% glacial acetic acid (**A**) and CH/PVP nanofiber (25/75 wt. %) with a weight ratio of 90 % (*v*/*v*) in acetic acid (**B**).

**Figure 2:**
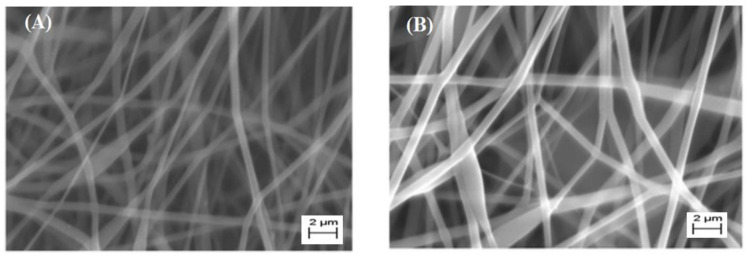

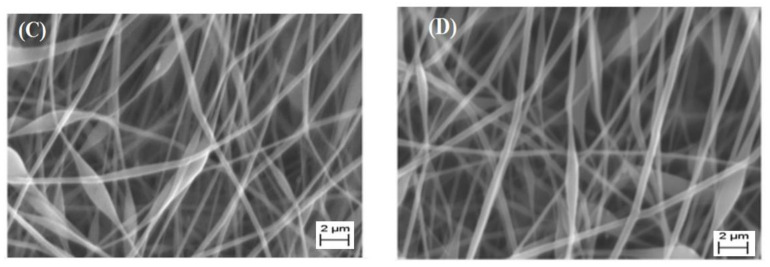
SEM images of electrospun CH/PVP nanofibers loaded with 10% 3β-cloro-5α-cholestano-2′-methyl-2-oxazoline (**A**), 3β-acetoxy-5α cholestano-2′-methyl-2-oxazoline (**B**), 3β-chloro-N-amido-5α-cholestano-aziridine (**C**) and 3β-acetoxy- N-amido-5α-cholestano-aziridine (**D**).

Electrospinning and the characterization of CH, as well as CH/PVP without and with synthesized 10% 3β-cloro-5α-cholestano-2′-methyl-2-oxazoline, 3β-acetoxy-5α cholestano-2′-methyl-2-oxazoline, 3β-chloro-N-amido-5α-cholestano-aziridine, and 3β-acetoxy- N-amido-5α-cholestano-aziridine, were investigated. A horizontal design setup for the electrospinning apparatus was used to obtain nanofibers from aqueous solutions. Figure 1A shows scanning electron microscope images of the electrospun pure chitosan. The micrograph shows that no real nanofibers were obtained, but rather, many beads formed. This was due to the fact that chitosan is soluble in most acids and the protonation of chitosan changes it into a polyelectrolyte in acidic solutions. The repulsive forces between ionic groups within a polymer backbone that arise due to the application of a high electric field during electrospinning restrict the formation of continuous fibers, and often produce beads. In addition, the formation of beads due to inadequate stretching of the filaments during the whipping of the jet resulted in a low charge density. To improve fiber formation and prevent the formation of beads, conductive polymers such as polyvinyl pyrrolidone were blended with the chitosan polymer solution. Figure 1B shows the surface morphology of electrospun nanofibers of chitosan and polyvinyl pyrrolidone blends (25/57 wt % respectively). The morphology reveals that nanofibers without beads formed. This was due to the stretching of the filaments, which increased with an increase of charge density or by the addition of conductive polymers. Furthermore, the SEM image clearly shows regular flat shapes with smooth surfaces with an average diameter in the range of 140–150 nm. Figure 2A–D shows the surface morphology of electrospun chitosan blend with polyvinyl pyrrolidone in the presence of 3β-cloro-5α-cholestano-2′-methyl-2-oxazoline (A), 3β-acetoxy-5α cholestano-2′-methyl-2-oxazoline (B), 3β-chloro-N-amido-5α-cholestano-aziridine (C) and 3β-acetoxy-N-amido-5α-cholestano-aziridine. The figures reveals that there was no effect on the surface smoothness or flatness of the electrospun nanofibers. In addition, the diameter of the electrospun nanofibers was slightly increased and there was no difference in the diameters of the loaded nanofibers. The diameter of loaded electrospun nanofibers ranged from 167 to 170 nm.

### 3.3. Swelling Behavior

**Figure 3:**
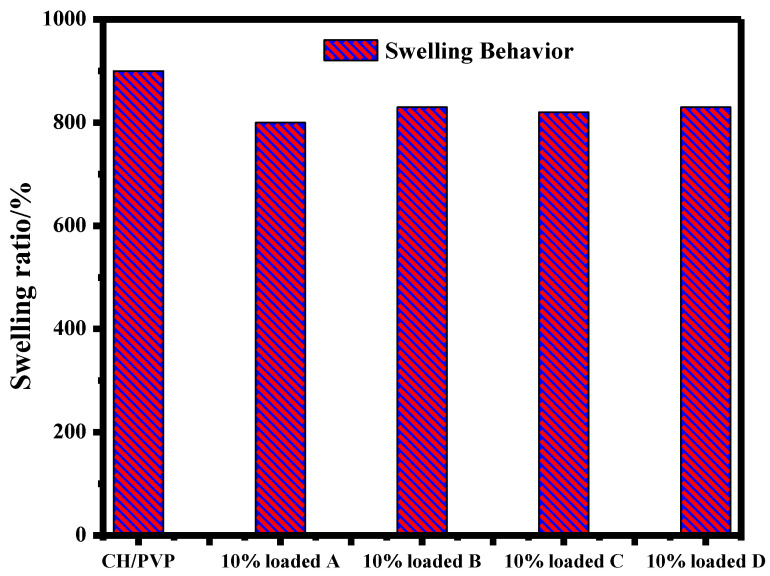
Swelling behavior of electrospun CH/PVP nanofibers loaded with 10% 3β-cloro-5α-cholestano-2′-methyl-2-oxazoline (A), 3β-acetoxy-5α cholestano-2′-methyl-2-oxazoline (B), 3β-chloro-N-amido-5α-cholestano-aziridine (C) and 3β-acetoxy-N-amido-5α-cholestano-aziridine (D).

To examine the ability of the chitosan-based nanofibers to absorb water, the swelling performance of the nanofibers was studied. The swelling ratio (%) correlates to the water absorption ability. The swelling ratio (%) of the electrospun nanofibers is detailed in Figure 3. It was observed that the swelling ratio of the CH/PVP mats did not decrease significantly following the incorporation of different steroidal oxazoline and aziridine compounds. In fact, all samples showed a high swelling ratio, i.e., 800–900%, which indicated the high-water absorption capability of the chitosan-based nanofibers.

### 3.4. Release Study

**Figure 4:**
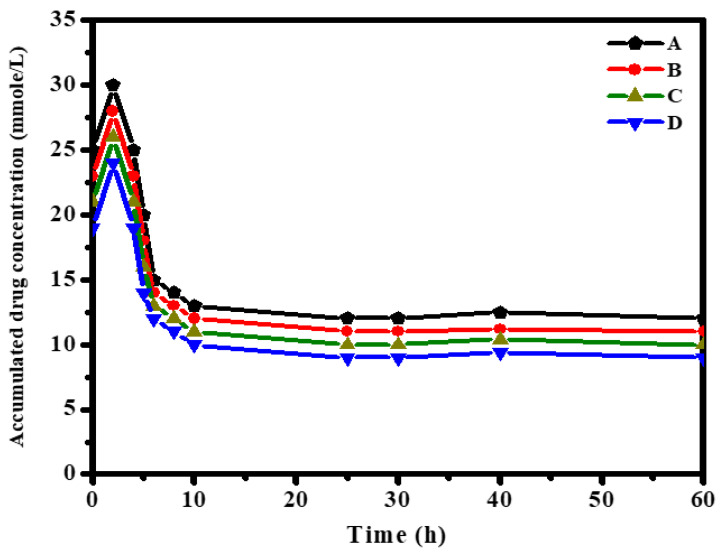
Release of 3β-cloro-5α-cholestano-2′-methyl-2-oxazoline (A), 3β-acetoxy-5α cholestano-2′-methyl-2-oxazoline (B), 3β-chloro-N-amido-5α-cholestano-aziridine (C) and 3β-acetoxy- N-amido-5α-cholestano-aziridine (D) from electrospun CH/PVP (25/75 wt. ratio) nanofibers.

Drug release from nanofibers should take place at the target location. The spatial control of drug carriage can be ensured by employing electrospun nanofibers at the targeted location via invasive or non-invasive means. Recently, the chronological control of drug release from electrospun nanofibers has been measured according to drug dissolution rates, drug dispersal rates, fiber diameters (lengths of diffusion barrier), drug physical-desorption rates, and/or polymer degradation/erosion rates [29,30,31,32,33,34]. Figure 4 displays the release of the steroidal derivatives-loaded electrospun nanofibers (samples A–D) in phosphate buffer saline. The data reveal that the release of drugs increased within the first 2 h due to the burst release behavior; after this time, the release rate decreased [35].

Drug release (A–D) was approximately constant between 10 and 30 h. The accumulated drug amount decreased with an increase in release time. The amount of released drug from the nanofiber sample showed the following decreasing order: A > B > C > D.

A further explanation for this behavior can be provided in terms of the charge density as well as the phenomenon of diffusion-controlled release. Electrostatic interactions among the incorporated drugs (samples A–D) within the electrospun CH/PVP nanofibers enhanced the charge density and the physical electrostatic binding between the protonated –NH_2_ groups in the chitosan-based matrix. Intercalated anions exist in the functional groups of the obtained drugs. However, the release amount showed the order A > B > C > D in the first 2 h. This conceivably resulted from the fact that samples C (~26 mmol/L) and D (~24 mmol/L) had greater charge density than samples A (~30 mmol/L) and B (~28 mmol/L), as shown through in bulk characterizations above. This higher charge density brought about stronger interactions with the Chitosan-based matrix. This can have the effect of increasing the energy barrier to release, further slowing the release rate [36].

Moreover, the diffusion-controlled release factor can also affect the behavior of the illustrated drugs. This performance can perhaps be attributed to the diffusion path length of the drug molecules within the release milieu [37]. A directly proportional relationship between the release rate and the diffusion coefficient [38], in which increasing the diffusion coefficient enhances the release rate, was observed for oxazoline derivatives, which are slightly smaller than aziridine derivatives. Thus, oxazoline derivatives were capable of diffusing out of the CH/PVP nanofibers more rapidly.

**Figure 5:**
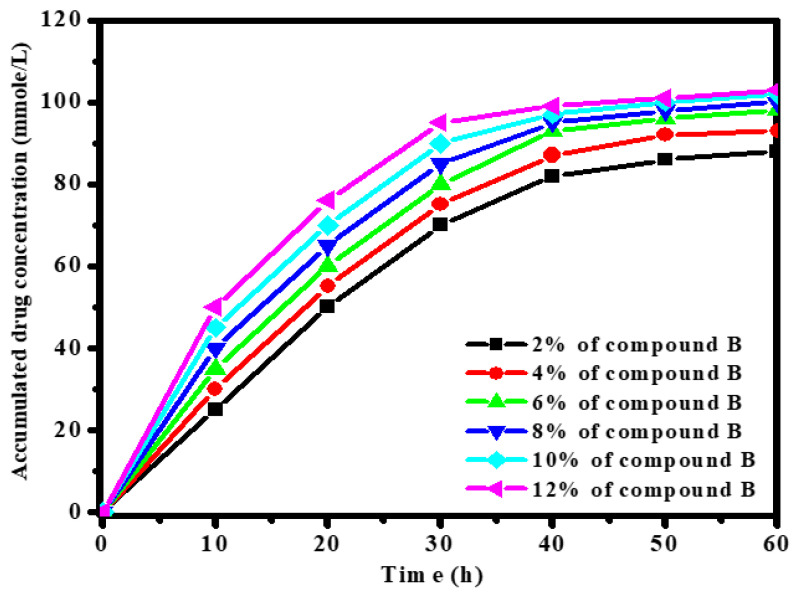
Effect of 3β-Acetoxy-5α-cholestano-2′-methyl-2-oxazoline (B) concentration [2,4,6,8,10,12] % on release rate.

Figure 5 shows the effect of drug accumulated concentration inside the electrospun nanofibers on the release rate. The data reveal that the release of drug B increased with increasing concentration inside the nanofibers. This was due to the fact that the greater drug charge formed more pores inside the fabricated fibers as drug molecules leaked into the medium, which facilitated the diffusion of drug B molecules to the release solution. As shown, the nanofibers achieved an almost constant rate of release between 30–60 h, when a slight plateau was reached. This behavior was attributed to the fact that a drug charge greater than 12 mmol will form more pores inside the fabricated fibers as drug molecules leak into the medium, which facilitates the diffusion of the drug molecules to the release solution. The loading behavior of the drug sample as a function of time occurred in two stages. The first stage displayed a strong increase in release rate due to the accessibility of open pores inside the fabricated fibers. This was followed by the second stage, i.e., a slight increase in drug concentration and steady release rates as the number of the free active sites increased with time [39].

To gain a deep understanding of the drug release mechanism, the Ritger-Peppas equation was applied to the release data of drug sample B (12%) within 30 h. The equation is as follows [35]:

(3) $$\frac{M_{t}}{M_{\infty }}=kt^{n}$$

where the fraction of drug A released at time t is represented by $\frac{M_{t}}{M_{\infty }}$ and the parameters *k* (rate constant) and exponent n will be given by the linear relation of log (*M_t_/M*∞) against log time (h), that could provide information about the essential release mechanisms.

After plotting this relationship, the intercept and slope values were calculated. Then, transport exponent n was estimated to be ~0.52 with an R^2^ value of 0.986. This value of *n* was between 0.5 and 1, considering the release mechanism as irregular (non-Fickian diffusion, which is a mixture of diffusion and polymer chain relaxation). Next, it was declared that drug release by the solvent diffusion and relaxation of the polymeric chains occurred consecutively. This was due to the dominant effect of the diffusion process with the influence of the relaxation process [40].

### 3.5. Antibacterial Activity

Next, we directed our attention to the antibacterial properties of the newly synthesized oxazoline and aziridine steroidal derivatives-loaded CH/PVP electrospun nanofibers. The antimicrobial properties were assessed against two bacterial strains, namely *Staphylococcus aureus* (gram-positive) and *Escherichia coli* (gram-negative). The respective diameters of the inhibition zone (in mm) of the nanofibers are listed in Table 5.

The nanofibers generally showed enhanced antibacterial activity against *S. aureus* compared to *E. coli*. This was probably due to the differential selective permeability of the bacteria cell wall. *Escherichia coli* (gram-negative) bacteria have two cytoplasmic membranes which hamper drug diffusion through the lipid membrane into the cytoplasm. On the other hand, *Staphylococcus aureus* (gram-positive) lacks an outer lipid membrane and has only a thin cytoplasmic membrane, which facilitates drug penetration (Figure S5).

The antibacterial activity of the electrospun nanofibers containing the synthesized steroidal aziridines was greater than that of the nanofibers containing steroidal oxazolines. The antibacterial activity of electrospun nanofibers containing 3β-cloro-5α-cholestano-2′-methyl-2-oxazoline and 3β-chloro-*N*-amido-5α-cholestano-aziridine were superior to those containing 3β-acetoxy-5α-cholestano-2′-methyl-2-oxazoline and 3β-acetoxy-*N*-amido-5α-cholestano-aziridine. In this context, the electrospun CH/PVP nanofibers containing 3β-chloro-*N*-amido-5α-cholestano-aziridine and those containing 3β-acetoxy-*N*-amido-5α-cholestano-aziridine were the most active, with A% of 91 and 104 % in case of *S. aureus*, and 52% and 61% in case of *E. coli*, respectively. On the other hand, the electrospun CH/PVP nanofibers containing 3β-cloro-5α-cholestano-2′-methyl-2-oxazoline and those containing 3β-acetoxy-5α cholestano-2′-methyl-2-oxazoline exhibited moderate antibacterial activities, with A% of 48 and 64 % against *S. aureus*, and 26 and 35 % against *E. coli,* respectively. Interestingly, the electrospun CH/PVP nanofibers containing 3β-acetoxy-*N*-amido-5α-cholestano-aziridine were even more potent than ampicillin (inhibition zone diameter = 22 vs. 21 mm, respectively) against *S. aureus*. These results indicate that the steroidal derivative had been released from CH/PVP electrospun nanofiber and was effective against *S. aureus*. Indeed, these results encorage further investigations employing a more expansive arsenal of bacterial strains.

## 4. Conclusions

Steroidal oxazolines and aziridines were synthesized from steroidal epoxides with acetamide, benzamide, and semicarbazide. The structures of these compounds were established by elemental analysis, FTIR, ^1^H NMR, and mass spectra analysis. An electrospun chitosan blend with polyvinyl pyrrolidone nanofibers loaded with synthesized steroidal derivatives of oxazolines and aziridines was fabricated by an electrospinning process. The surface morphology and diameter of electrospun nanofibers were characterized with SEM. The obtained data revealed that nanofibers without beads had been formed with regular flat shapes with smooth surfaces, with an average diameter size in the range of 140–150 nm. Furthermore, there was no effect on the surface smoothness or flatness of the loaded electrospun nanofibers. The diameter of loaded electrospun nanofibers increased slightly, and there was no difference in the diameters of the loaded nanofibers. The diameters of obtained loaded electrospun nanofibers ranged from 167 to 170 nm. Furthermore, the swelling ratio of the CH/PVP mats did not decrease significantly following the incorporation of different synthesized steroidal epoxides. All samples showed a high swelling ratio, i.e., between 800–900%, which indicated a high water absorption capability. The release of drugs increased within the first 2 h, which was a consequence of the high charge density and the diffusion-controlled release mechanism. Moreover, the release rate was affected by the concentration of the drug inside the electrospun nanofibers. The drug release mechanism of derivative B over CH/PVP revealed both the diffusion and polymeric relaxation processes in light of the assessed value of exponent n as the transport factor using the Ritger-Peppas kinetic model, with a dominant effect of solvent diffusion. 

Finally, the electrospun CH/PVP did not display antibacterial activity against *E. coli,* but had slight activity toward S. aureus. Both bacteria were sensitive to steroidal oxazoline and aziridine-loaded chitosan/PVP electrospun nanofibers.

## Figures and Tables

**Table 1:** FTIR, ^1^HNMR, and mass analyses of 3β-Chloro-5α-cholestano-2′-methyl-2-oxazoline (**V**).

Elution	Petroleum Ether: Ether (10:1).
% Yield	79.58 %, m.p. 110 °C.
%C_29_H_48_NOCl requires	C, 75.48; H, 10.41; N, 3.03; O, 3.47 Cl, 7.59.
FTIR	ν max 1697 cm^−1^ (C=N), 1334 cm^−1^ (C–N) and 1100 cm^−1^ (C–O), 3649 cm^−1^ (N–H), 2962 cm^−1^ (C–H) and 717 cm^−1^ (C–Cl).
^1^HNMR (CDCl_3_)	δ 5.50 (1H, dd, H-6β), 3.90 (multiplet, 1H, H-3α) 2.15 (s, 3H,CH_3_–C=N), 1.02 (C10–CH_3_), 0.65 (C13–CH_3_), 0.98 and 0.88 (side chain methyl proton).
Mass	*m*/*z* 461 (M+), *m*/*z* 404, *m*/*z* 57.

**Table 2:** FTIR, 1HNMR, and mass analyses of-acetoxy-5α-cholestano-2′-methyl-2-oxazoline (**VI**).

Elution	Petroleum Ether: Ether (10:1).
Yield%	74.91%, m.p. 110 °C
%C_31_H_51_NO_3_ requires	C, 6.70; H, 10.51; N, 2.88; O, 9.91.
FTIR	ν max 1735 cm^−1^ (CH_3_–COO–), 1685 cm^−1^ (C=N), 1360 cm^−1^ (C–N), 1270 cm^−1^ and 1030 cm^−1^ (C–O).
^1H^NMR (CDCl_3_)	δ 5.40 (multiplet, 1H, H-6β), δ4.10 (multiplet,1H, H-3α) δ2.3 (s, 3H, CH_3_–C=N), δ2.10 (s,CH_3_–COO–), δ1.02 (C10–CH_3_), δ0.69 (C13 –CH_3_), δ 0.94 and 0.86 (side chain methyl proton).
Mass	*m*/*z* 485 (M+), *m*/*z* 368/369, *m*/*z* 57 & *m*/*z* 59.

**Table 3:** FTIR, 1HNMR and mass analyses of 3β-chloro-N-amido-5α-cholestano-aziridine (**VII**).

Elution	Petroleum Ether: Ether (10:1).
Yield%	76.2 %, m.p. 132 °C.
% C_28_H_47_N_2_OCl requires	C, 78.97; H, 9.68; N, 2.55; O, 8.77. Cl 7.57
FTIR	V max 3564 cm^−1^ (–N–H), 1697 cm^−1^ (–CO–NH), 1380 cm^−1^ (C–N) and 717 cm^−1^ (C–Cl).
^1^H-NMR (CDCl_3_)	5.2 (S, 2H, exchangeable with D_2_O–NH_2_), 3.60 (multiplet 1H, H-6β, axial), 4.5 (doublet 1H, H-3α, axial). The methyl protons gave signals at δ 1.1 (C10–CH_3_), 0.70 (C13–CH_3_), 0.95, and 0.88 (side chain methyl protons).
Mass	*m*/*z* 462/464 (M+), *m*/*z* 406 and 58/57.

**Table 4:** FTIR, 1HNMR and mass analyses of 3β-acetoxy-N-amido-5α-cholestano aziridine (**VIII**).

Elution	Petroleum Ether: Ether (10:1).
Yield%	71.83 %, m.p. 177–178 °C.
%C_30_H_50_N_2_O_3_ requires	C, 74.07; H, 10.28; N, 5.67; O, 9.87.
FTIR	ν max 3548 (N–H), 1697(–COO–CH_3_), 1651 (NH–CO–), 1396 (C–N), 1026 (C–O).
^1^HNMR (CDCl_3_)	δ 5.3 (s, 2H, exchangeable with D_2_O–NH_2_) 4.3 doublet, 1H, H-3 α-axial), 3.90 (multiplet centered at, 1H, H-6β), 2.5 (s, 3H, methyl proton), 1.1 (C10–CH_3_), 0.74 (C13–CH_3_), 0.97 and 0.85 (side chain methyl proton).
Mass	*m*/*z* 484/486 (M+), *m*/*z* 368/369, *m*/*z* 57 (NH_2_–CO–N), *m*/*z* 59 (CH_3_–COO).

**Table 5:** Antimicrobial properties of the electrospun CH/PVP nanofibers.

Samples ^b^	*E. coli*		*S. aureus*	
	Diameter (mm) ^a^	% Activity Index	Diameter (mm) ^a^	% Activity Index
Electrospun CH/PVP nanofibers	0	-	0	-
Electrospun CH/PVP nanofibers containing 3β-cloro-5α-cholestano-2′-methyl-2-oxazoline	6	26	10	48
Electrospun CH/PVP nanofibers containing 3β-acetoxy-5α cholestano-2′-methyl-2-oxazoline	8	35	14	64
Electrospun CH/PVP nanofibers containing 3β-chloro-N-amido-5α-cholestano-aziridine	12	52	20	91
Electrospun CH/PVP nanofibers containing 3β-acetoxy- N-amido-5α-cholestano-aziridine	14	61	22	104
Ampicillin ^b^	23	-	21	-

^a^ Disk papers (5 mm) were soaked in 20 μM of the nanofiber solution in DMSO; ^b^ The activity was measured after one day of incubation with the nanofibers. Ampicillin was used as the reference compound, and all experiments were replicated.

## Data Availability

The raw/processed data generated in this work are available upon request from the corresponding author.

## Supplementary Materials

The following supporting information can be downloaded at: https://www.mdpi.com/article/10.3390/polym14102094/s1. Figure S1: Spectral analysis of 3β-Chloro-5α-cholestano-2’-methyl-2-oxazoline (**V**). Figure S2: Spectral analysis of 3 β-acetoxy-5α-cholestano-2’-methyl-2-oxazoline (**VI**). Figure S3: Spectral analysis of 3β-chloro-N-amido-5α-cholestano-aziridine (**VII**). Figure S4: Spectral analysis of 3β-acetoxy-N-amido-5α-cholestano aziridine (**VIII**). Figure S5: Antibacterial activity of CH/PVP nanofibers and (a) electrospun CH/PVP nanofibers containing 3β-cloro-5α-cholestano-2’-methyl-2-oxazoline, (b) electrospun CH/PVP nanofibers containing 3β-acetoxy-5α cholestano-2’-methyl-2-oxazoline, (c) electrospun CH/PVP nanofibers containing 3β-chloro-N-amido-5α-cholestano-aziridine, and (d) electrospun CH/PVP nanofibers containing 3β-acetoxy- N-amido-5α-cholestano-aziridine against *S. aureus* and *E. coli*.
