# Supplementary material for: Fabrication of Chitosan Nanofibers Containing Some Steroidal Compounds as a Drug Delivery System
Source: Polymers (Basel). 2022 May 20;14(10):2094. doi: 10.3390/polym14102094 (PMC9146270; doi:10.3390/polym14102094)
Supplement: Supplementary file 1 [file polymers-14-02094-s001.zip › polymers-1717740-SI.pdf]

Supporting Information (SI)

Drug Delivery System Based Chitosan Nanofibers Containing Steroidal Compounds

Mohamed Gouda[1,2*], Mai M. Khalaf [1,2,3], Saad Shaaban[1,3,4], Hany M. Abd El-Lateef [1,2,3**]

[1] *Al Bilad Bank Scholarly for food security in Saudi Arabia, the deanship of scientific research, the vice presidency for graduates students and scientific research, King Faisal University, Al-Ahsa 31982, Saudi Arabia*

[2] *Department of Chemistry, College of Science, King Faisal University, Al-Ahsa 31982, Saudi Arabia*

[3] *Department of Chemistry, Faculty of Science, Sohag University, Sohag 82524, Egypt*

[4] *Department of Chemistry, Faculty of Science, Mansoura University, 35516 Mansoura, Egypt*

* Corresponding author: Email: ***mgoudaam@kfu.edu.sa** (M. Gouda) ,

****hmahmed@kfu.edu.sa** (H M Abd El-Lateef)

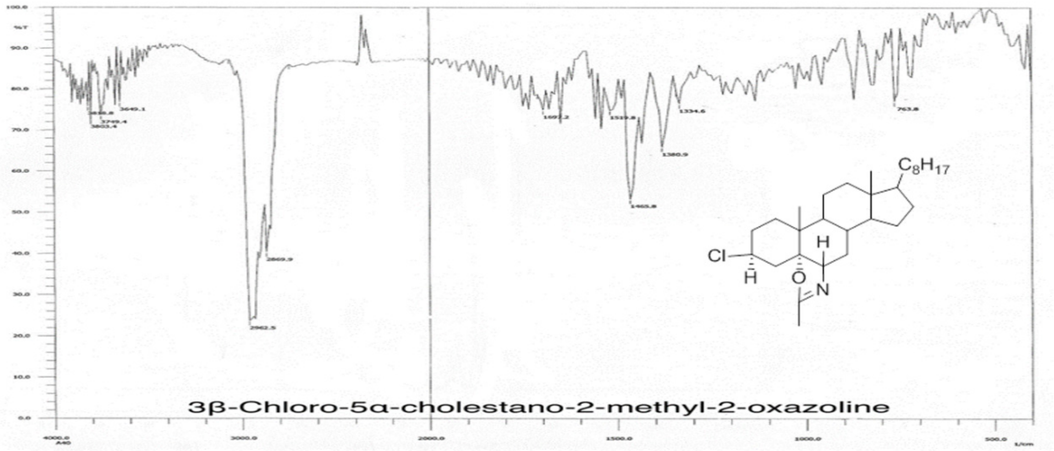

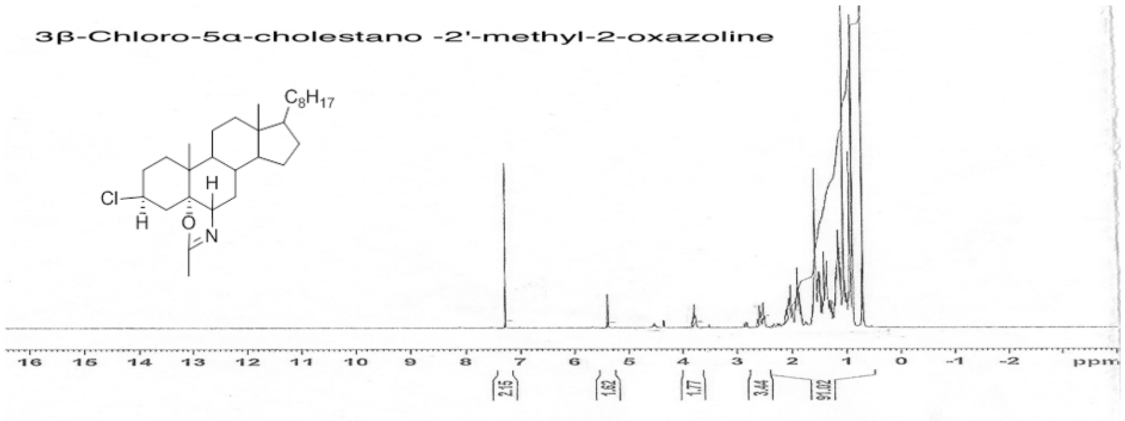

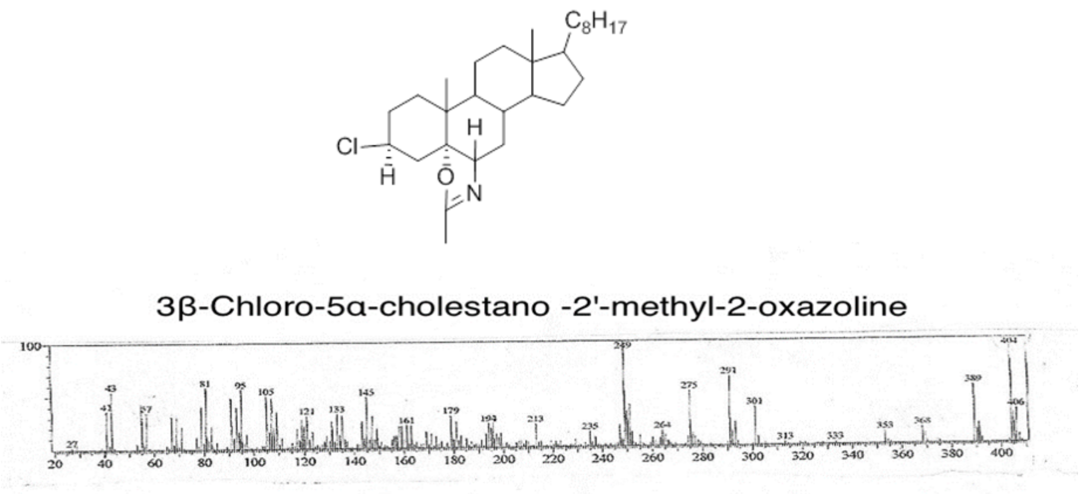

Figure S1: spectral analysis of 3β-Chloro-5α-cholestano-2'-methyl-2-oxazoline (V).

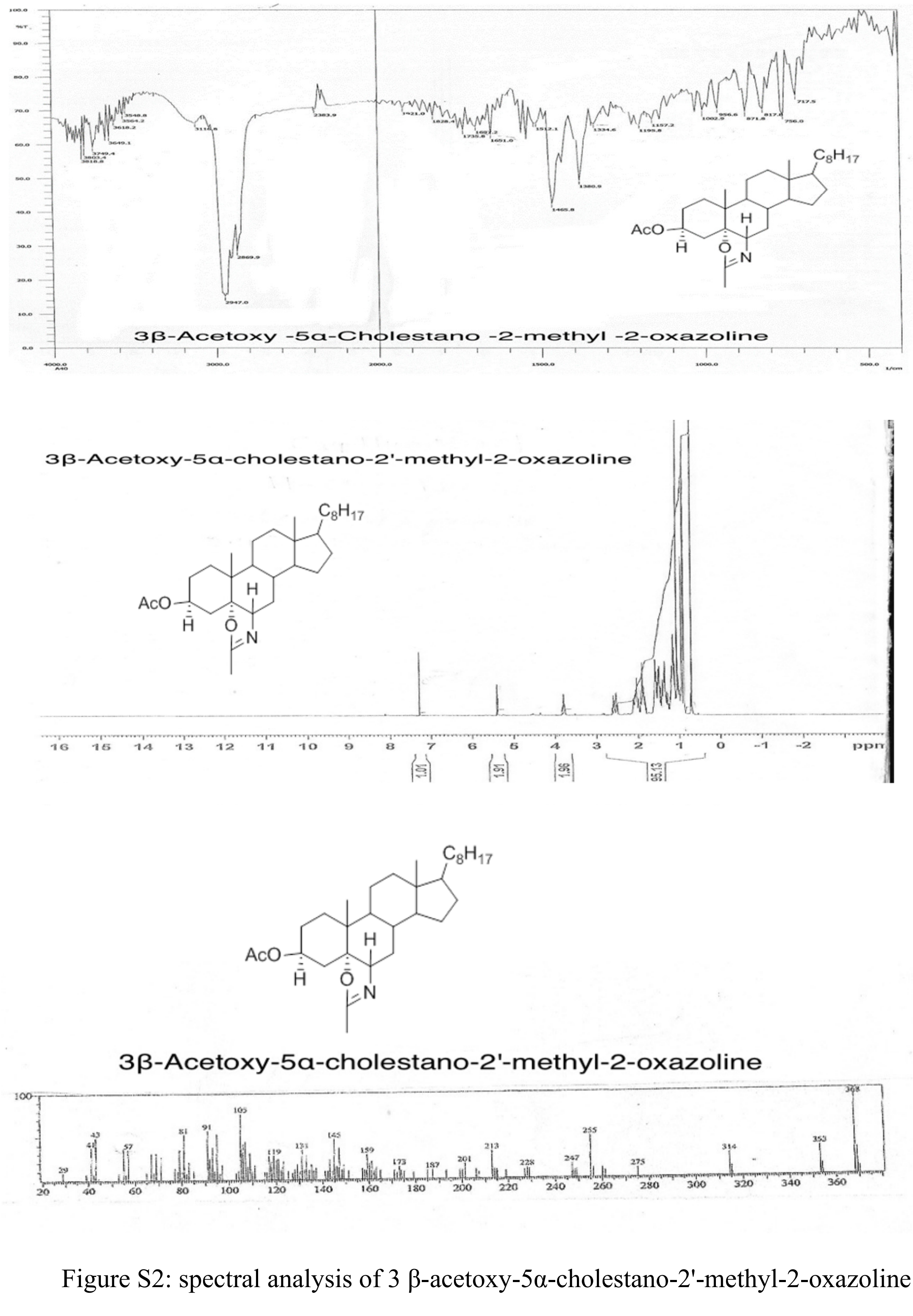

Figure S2: spectral analysis of 3 β-acetoxy-5α-cholestano-2'-methyl-2-oxazoline (VI).

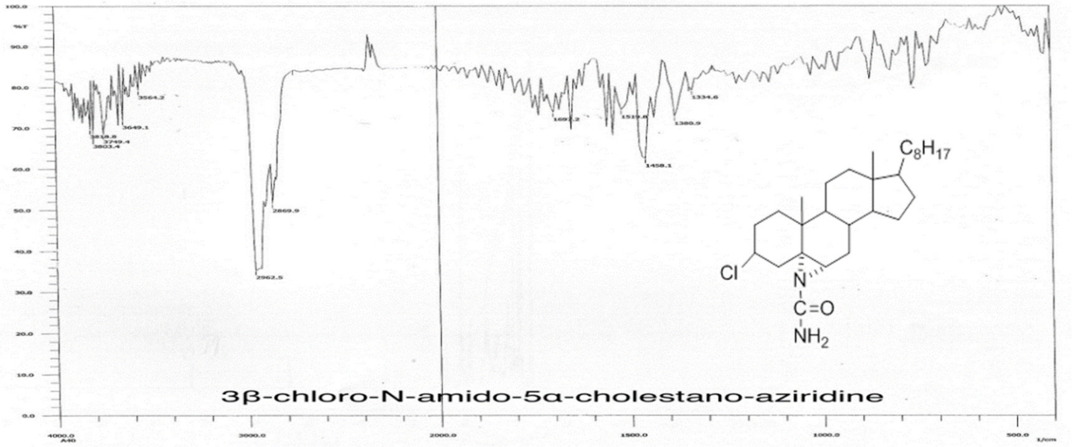

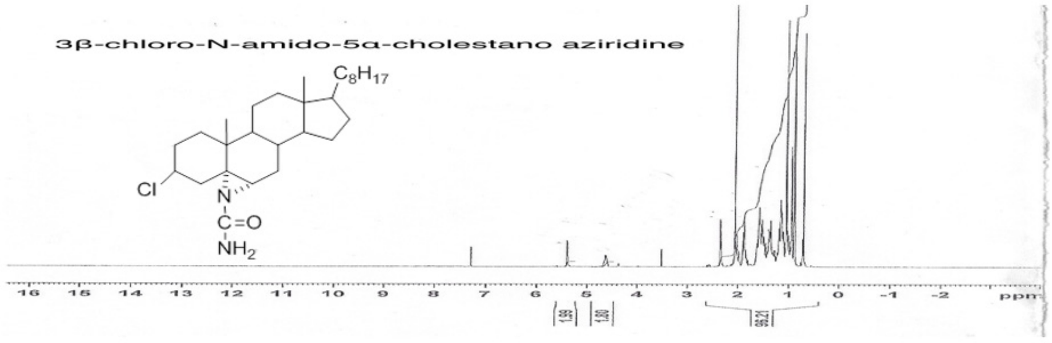

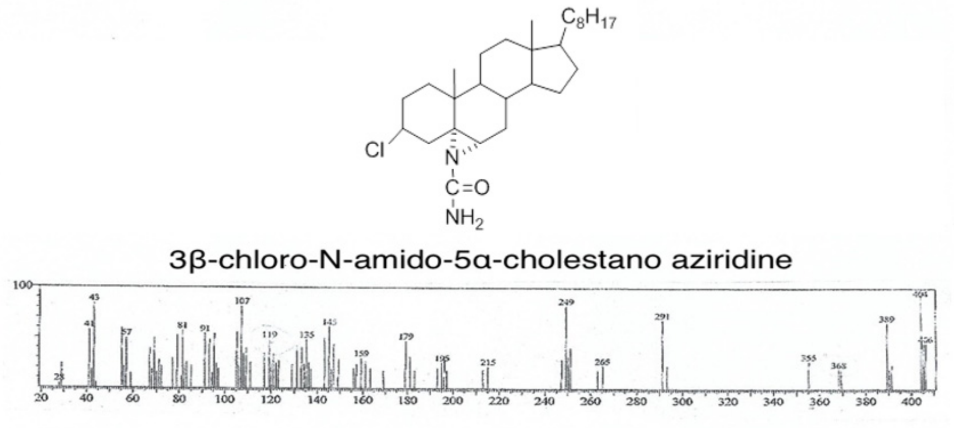

Figure S3: spectral analysis of 3β-chloro-N-amido-5α-cholestano-aziridine (VII).

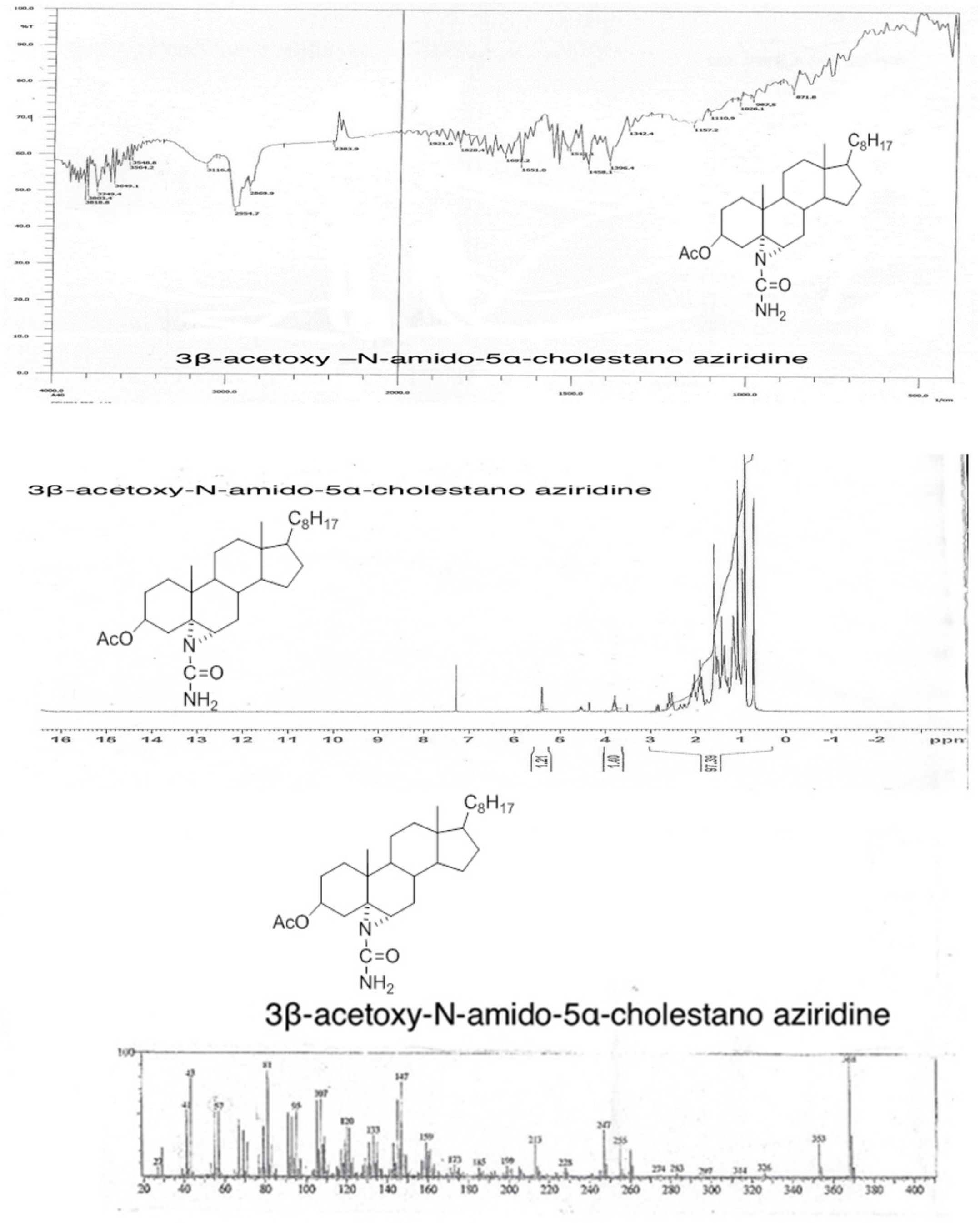

Figure S4: spectral analysis of 3β-acetoxy-N-amido-5α-cholestano aziridine (VIII).

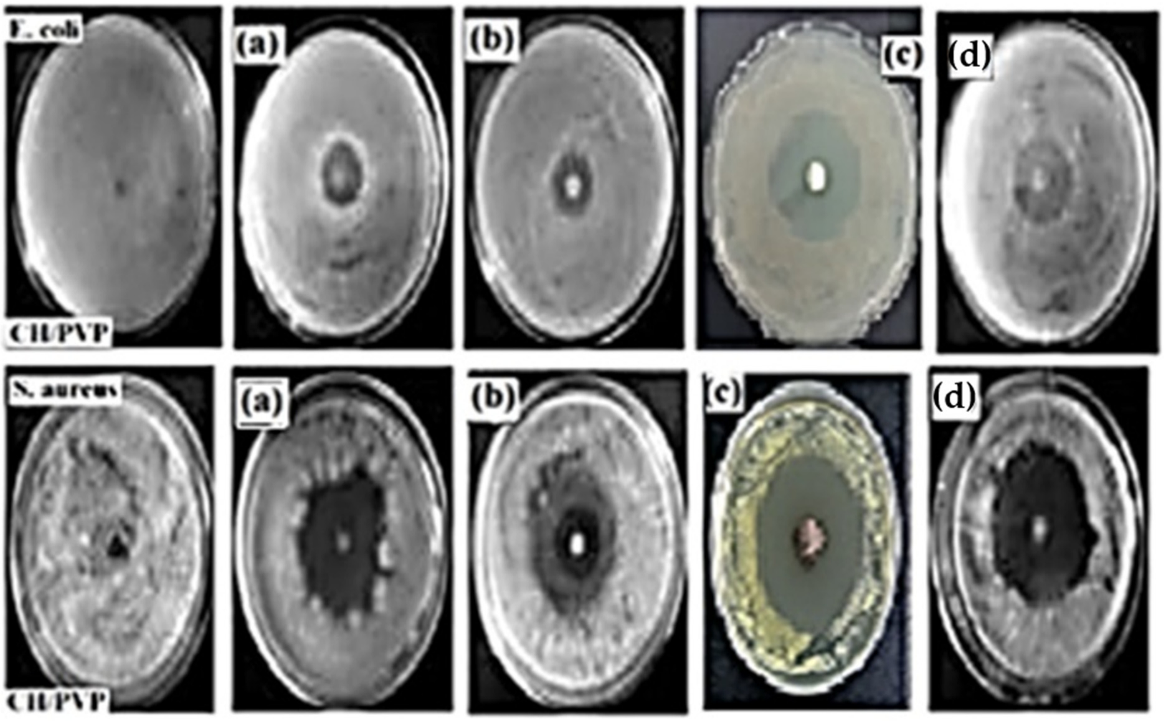

Figure S5. antibacterial activity of CH/PVP nanofibers and 3β-cloro-5α-cholestano-2'-methyl-2-oxazoline (**a**), 3β-acetoxy-5α cholestano-2'-methyl-2-oxazoline (**b**), 3β-chloro-N-amido-5α-cholestano-aziridine (**c**) and 3β-acetoxy- N-amido-5α-cholestano-aziridine (**d**) loaded- CH/PVP electrospun nanofibers against *S. aureus* and *E. coli*.
